# Thuja Occidentalis

**Published:** 1891-07-25

**Authors:** 


### THERAPEUTICS.

### THUJA OCCIDENTALIS.

The Thuja Occidentalis is a coniferous tree, which is described in the U.S. Dispensatory as attaining to about fifty feet in height and three feet in diameter, and growing in swampy grounds in Pennsylvania northward and westward, often forming extensive "cedar swamps." The part used is the leaf or the small twigs with the leaves, which have an agreeable balsamic odour, especially when rubbed, and a strong, balsamic, camphoraceous bitter taste, and are flattish, two-edged, with the scale-like leaves oppressed and closely imbricate in four rows, rhombic ovate, obtusely pointed. Kawalier discovered in the leaves a peculiar crystallisable colouring principle, which he names thujin, and to which he gives the formula $C_{20}\ H_{22}\ O_{12}$. It is of a citron-yellow colour and an astringent taste, soluble in alcohol, inflammable, and when its alcoholic solution is heated with dilute hydrochloric acid or sulphuric acid it splits up.

As regards its medical properties, it has been used in the form of a decoction in intermittent fever, and, according to Schœpf, in coughs, fevers, scurvy, and rheumatism. Its proper sphere is, however, limited to new growths, chiefly warts. A recent writer credits it with "the remarkable property of causing the disappearance, in a very short time, of all kinds of vegetations and warty growths by its internal administration. Many successful cases are reported by French physicians; in fact, it is said to seldom or never fail. It is given in the form of tincture, from ʒss. to ʒi., two or three times a day. Its action is regarded as quite phenomenal in this direction. Among others, Dr. Constantine Paul reports the cure within fourteen days of non-syphilitic warts (labial). It is said to be equally serviceable in the case of condylomata and other similar growths." Now, our experience of this remedy enables us to say that, valuable as thuja undoubtedly is in certain conditions, such fulsome praise will only lead to disappointment, and perhaps cause those who make use of it to overlook the service rendered by thuja in properly selected cases. In the following note Dr. Baratoux, of Paris, very fairly indicates the kind of cases in which this beneficial action may be expected. In tumours of the ear, nose, mouth, pharynx, and larynx, especially those of a neoplasmic character in the latter organs, the first effect noticed is the rapid decrease and disappearance of the fœtid smell characterising the mucous and purulent discharges. The next is a decrease in the quantity of the secretions, and the third, usually noticeable after about twenty days, a reduction in the size of the vegetations. In nose and ear papilloma complete cure may be obtained. But whenever the tumour is a complex one, only partial relief is secured, the neoplasm falling off as to size, but never disappearing altogether, while sarcomata and carcinomata remain seemingly unaffected by the treatment. And, finally, in epithelioma, Dr. Baratoux recommends thuja as very efficient in checking the evolution of the disease, and especially lessening the offensive odours peculiar to the ulcerative period of cancer, and states that thuja in epithelioma of the larynx is only a palliative, which may delay, but cannot obviate the necessity of a surgical operation, which is the only hope of cure. Coculet recommends the employment of the tincture of thuja to destroy the fleshy vegetations which cause the irritation in the region of the cannula after tracheotomy, and quotes two cases in support of his suggestion. Cheron's experience of the use of thuja in epithelioma of the cervix, extending over several years, led him to say that in a certain number of cases he uses the tincture internally only; in a second class externally only; and in the third class he uses it both internally and externally. He found that in vegetations covered with epidermis, especially those which have a semicorneal consistence, are not affected by the internal administration of thuja, so far as their disappearance is concerned, though they do diminish in size and consistence. Vegetations developed on mucous membranes, however, disappear completely when thuja is given internally; this has been abundantly shown in a great number of cases. In a word, the epithelial hyperplasias of the vegetations yield to the internal employment of thuja, while the epidermic hyperplasias do not.

The remedy should generally be used both internally and externally. Internally the tincture should be given in doses of 10 to 60 drops, three or four times daily, and externally it should be applied to the parts in the form of compress of the tincture diluted with water.

* *British Medical Journal.* Vol. I., 1891, p. 404. Pract. May, 1891.

